# Effects of planting density on the growth of *Xanthoceras sorbifolium*, soil nutrients, and microbial community assembly processes

**DOI:** 10.3389/fmicb.2026.1749267

**Published:** 2026-03-30

**Authors:** Yuexin Zhang, Yunxia Ma, Xiuzhi Ma, Cuiwei Li, Haibing Wang

**Affiliations:** 1 State Key Laboratory of Water Engineering Ecology and Environment in Arid Area, Inner Mongolia Agricultural University, Hohhot, China; 2 College of Forestry, Inner Mongolia Agricultural University, Hohhot, China; 3 College of Desert Governance, Inner Mongolia Agricultural University, Hohhot, China

**Keywords:** microbial community, soil microorganisms, soil nutrients, stand density, *Xanthoceras sorbifolium*, planting density

## Abstract

Comprehending the role of stand density in soil properties and microbial communities is crucial for optimizing forest ecosystem functions. However, the relationship between them and how they respond to key environmental drivers at different planting densities remains unclear. In this study, we investigated the growth indices, soil properties, as well as soil microbial community composition and diversity of two *Xanthoceras sorbifolium* provenances (YC and KL) under three planting densities (low, medium, and high). Results indicated: (1) soil nutrient levels decreased with increasing stand density, while microbial community diversity was highest at medium density, followed by low and high density. Low planting density was more favorable for *X. sorbifolium* growth. (2) Microbial co-occurrence network stability was highest under medium and low densities. The assembly of bacterial communities was primarily governed by deterministic processes, whereas fungal community assembly was predominantly stochastic. (3) Soil total phosphorus (TP) and available potassium (AK) were the main drivers affecting microbial communities and their structures. (4) Compared with high-density soil, there were more abundant functional communities of bacteria and fungi in low- and medium-density soils, showing better adaptability to environmental changes and disturbances. These results will provide a scientific basis for the management optimization of *X. sorbifolium* plantations and are of great significance for the sustainable development of forest ecosystems.

## 1 Introduction

Stand density refers to the number of standing trees per unit area of forest land. As a key factor in forest management, it plays a significant role in maintaining the balance of forest ecosystems (Xie and Zhang, 2018). Specifically, higher stand density increases the competition for light and soil, inhibits the growth of tree crowns, intensifies the differentiation between tree height and diameter at breast height, and alters the growth environment and spatial distribution of the stand (Ao et al., 2016). Lower stand density reduces the density of trees per unit area, resulting in waste of forest land and a decrease in stand productivity and biodiversity (Chivhenge et al., 2024). Ideally, an appropriate stand density can promote the formation of a sound stand structure, improve the microenvironment under the canopy, and enhance nutrient cycling, thus optimizing the resource allocation for individual trees, increasing tree productivity, and ultimately maximizing the ecological benefits of forests (Xu et al., 2025).

Soil serves as the foundation of forest growth. It performs a series of ecological functions: providing nutrients and water, decomposing and synthesizing humus, retaining water, preventing erosion, and regulating climate (Büntgen et al., 2019). Soil microorganisms, the most widespread and active component of the soil ecosystem, drive important underground ecological processes, such as material cycling and nutrient transformation, acting as regulators and catalysts (Zhang J. et al., 2021; Zhang Y. X. et al., 2021; Peco et al., 2017). The community structure and functional diversity of soil microorganisms can directly affect soil nutrients, and can also form complex interaction networks with plants through mycorrhizal symbiosis and metabolic product exchange (Yang et al., 2014). Stand density, as a core parameter in artificial forest management, may lead to changes in light, temperature, and humidity, directly affecting the soil microenvironment, which, in turn, influences the structure and function of soil microbial communities (Peng et al., 2020). An excessively high stand density may exacerbate the competition between plant species and lead to an increase in soil pathogens, being detrimental to plant growth (Burton et al., 2010; Zhang et al., 2020). In addition, high forest canopy closure and poor light, heat, and water conditions may cause significant competition among microbial communities, influencing biomass stability and soil nutrient cycling. Studies have shown that in low-density stands, where resources are relatively abundant and habitat heterogeneity is high, deterministic processes typically dominate microbial community assembly. However, as stand density increases, intensified interspecific competition may lead to a narrowing of niche breadth, potentially enhancing the contribution of stochastic processes like random dispersal and drift to community structure. This reflects the complexity and unpredictability of biological interactions under high-density stress (Jiao et al., 2020). Therefore, comprehending the relationship between artificial forest density and soil microbial communities is of particular significance for the productivity and sustainable development of artificial forests. Existing research on artificial forest density mainly investigates its impact on tree performance, the effects of different planting densities on growth, health, and overall forest productivity (Selvalakshmi et al., 2022; Wan et al., 2023). However, the interactions between artificial forest density, tree species characteristics, soil properties, and microbial community dynamics have not been sufficiently explored.

*Xanthoceras sorbifolium*, a rare woody oil tree species unique to northern China, with strong ecological adaptability and stress resistance, can be used as a raw material for biodiesel (Ma et al., 2025). As the *X. sorbifolium* industry has gained increasing attention, the area under artificial cultivation has continued to expand. Currently, there are large *X. sorbifolium* plantations in regions such as Ningxia and Inner Mongolia in China. However, studies show that inappropriate planting densities have led to slow growth and reduced yields in artificial Wenguan fruit forests. Excessively high planting densities will result in competition among individuals for underground nutrients and above-ground canopy growth space, with soil fertility also suffering a sharp decline (Wang et al., 2021). To address this knowledge gap, we investigated the coupled relationship between soil microbial communities, plant growth characteristics, and soil nutrients in *X. sorbifolium* artificial forests of different densities in Tongliao City, Inner Mongolia, China, as well as the primary factors driving changes in microbial communities. We hypothesized that planting density regulates the growth of *X. sorbifolium* and soil physicochemical properties, thereby influencing soil microbial community composition and assembly processes. Specifically: (1) low and medium densities enhance soil nutrient availability (particularly total phosphorus and available potassium) and promote plant growth, while high density leads to nutrient depletion and reduced microbial diversity; (2) soil microbial community structure is primarily driven by total phosphorus and available potassium, with bacterial communities governed by deterministic assembly processes and fungal communities by stochastic assembly processes; and (3) microbial co-occurrence networks exhibit higher stability under low and medium densities compared to high density.

## 2 Materials and methods

### 2.1 Study location

The research site is situated at the *X. sorbifolium* cultivation base in Kailu County, Tongliao City, Inner Mongolia Autonomous Region, China (120°25′-120°26′E, 43°14′-43°19’N). The region exhibits a temperate continental semi-arid monsoon climate with distinct seasons. Annual average precipitation stands at 338.3 mm, with an annual mean temperature of 5.9 °C. The frost-free period lasts 148 days, and the area enjoys ample sunshine, recording 3,095 h of annual sunshine duration. The terrain is flat, and the soil is uniformly sandy loam.

This study utilized a 15-year-old *X. sorbifolium* density experimental forest established by Beijing Forestry University within this base. Under identical management conditions, the experimental forest was planted at three densities: low (approximately 1,050 trees/ha), medium (approximately 1,425 trees/ha) and high (approximately 2,350 trees/ha). Each density included two provenances: Yinchuan (YC) and Kailu (KL). The YC provenance (Yinchuan, Ningxia) was collected from natural/planted forests of *X. sorbifolium* in Yinchuan City (38°25′-38°35′N, 106°10′-106°20′E), with the original germplasm traceable to the germplasm repository preserved by the Ningxia Forestry Institute. The KL provenance (Kailu, Inner Mongolia) was collected from planted forests of *X. sorbifolium* in Kailu County (43°14′-43°19′N, 120°25′-120°26′E), which is the same location as the study site.

### 2.2 Growth measurement

Tree height (TH) was measured using a Vertex IV altimeter (Haglof, Sweden) with an accuracy of 0.01 m; diameter at breast height (DBH) was measured using a steel tape measure at a height of 1.3 m above ground level at the base of the trunk, with an accuracy of 0.01 cm; crown breadth (CB) was measured using an optical vertical instrument, with crown radius measurements taken along eight main cardinal directions.

### 2.3 Soil sample collection and physical and chemical property determination

In August 2024, soil sampling was conducted in forest stands with three density levels (low, medium, and high). Each density level included two provenances (YC and KL). For each density × provenance combination, six independent 20 m × 20 m quadrats were randomly established as replicates. Soil samples were collected from the 0–20 cm layer using a 5 cm diameter soil auger following a five-point sampling method within each quadrat. The soil from the five sampling points within each quadrat was thoroughly mixed to form one composite soil sample. After removing debris such as plant residues, the samples were sealed and transported to the laboratory. A total of 36 soil samples were collected, encompassing 3 density levels, 2 provenances, and 6 independent replicates. Each soil sample was divided into two parts: one part was immediately stored at −80 °C for soil microbial community analysis, and the other part was air-dried for soil physicochemical property analysis.

Soil pH was determined potentiometrically using a pH meter with a water-to-soil ratio of 2.5:1 (Wan et al., 2015); Total Nitrogen (TN) was determined by the Kjeldahl method (Bao, 2000); Total Phosphorus (TP) was determined by the molybdenum-antimony colorimetric method (De Rooij, 2004); Total Potassium (TK) was determined by the sodium hydroxide fusion–flame photometry method (De Rooij, 2004); Available Nitrogen (AN) was determined by the potassium persulfate oxidation method (Cheng et al., 2017); Available Phosphorus (AP) was extracted with NH4F and determined by the colorimetric method (Pfahler et al., 2020); Available Potassium (AK) was determined by atomic absorption spectrophotometry; Soil Organic Matter (SOM) was determined by the K₂Cr₂O₇-H₂SO₄ oxidation method (Lefroy et al., 1993).

### 2.4 DNA extraction and high-throughput sequencing

DNA was extracted from soil samples using the OMEGA Soil DNA Kit (M5635-02, Omega Bio-Tek, Norcross, GA, United States). The extracted DNA was diluted to a concentration of 10 ng μL^−1^ and stored at −20 °C for subsequent use as template in PCR. The bacterial 16S rRNA gene (V3–V4) was amplified using primers 338F (5’-ACTCCTACGGGAGGCAGCA-3′) and 806R (5’-GGACTACHVGGGTWTCTAAT-3′). The fungal ITS region was amplified using primers ITS1F (5’-CTTGGTCATTTAGAGGAAGTAA-3′) and ITS2 (5’-GCTGCGTTCTTCATCGATGC-3′). The resulting amplicons were purified following standard protocols and sequenced on an Illumina MiSeq PE300 platform by Shanghai Meiji Biotechnology Co., Ltd. (Shanghai, China). Raw paired-end reads were merged using FLASH (v1.2.11) and quality-filtered using fastp (v0.20.0). Denoising (to infer amplicon sequence variants, ASVs) and chimera removal were performed with the DADA2 plugin within QIIME 2 (v2023.2). Taxonomic classification of bacterial and fungal ASVs was conducted using the SILVA 138 (Quast et al., 2013) and UNITE 8.0 (Nilsson et al., 2019) databases, respectively. Any sequences identified as originating from archaea, mitochondria, or chloroplasts were removed, and the remaining high-quality ASVs were used for further analysis.

### 2.5 Statistical analysis

Data organization was performed using Microsoft Office 2019 software. Two-way ANOVA was performed to evaluate the main effects of planting density, provenance, and their density × provenance interaction. Duncan’s multiple range test was used for post-hoc comparisons (*α* = 0.05). Alpha diversity indices were calculated using the “vegan” package. Based on the Bray–Curtis distance matrix, principal coordinate analysis (PCoA) was performed via the “vegan” package, and permutational multivariate analysis of variance (PERMANOVA, executed using the adonis2 function with 999 permutations) was employed to examine overall differences in microbial community structure among different density groups. To construct co-occurrence networks, we used SPIEC-EASI to address the compositional nature of the sequencing data. Model selection was performed using the StARS method, and multiple testing was controlled using the Benjamini–Hochberg procedure (*q* < 0.05). Network topological parameters were calculated using the “igraph” package. To assess network stability, we performed bootstrap resampling (1,000 iterations) and node-removal robustness analysis. Network visualization was performed using Gephi 0.9.2. Community assembly processes were assessed by calculating the β-nearest taxon index (βNTI) using the “picante” and “ape” packages to infer the relative contributions of deterministic and stochastic processes to community assembly (Sheng et al., 2021). Prior to redundancy analysis (RDA), Variance Inflation Factor (VIF) analysis was conducted to assess multicollinearity among soil physicochemical factors. All factors exhibited VIF values below 10, indicating no severe multicollinearity; therefore, all variables were retained for subsequent analysis. Mantel tests were applied to evaluate the correlation between microbial community structure and soil nutrient content, and distance-based redundancy analysis (RDA) was further utilized to quantify the explanatory power of soil physicochemical properties on community structure. Additionally, the functional potential of bacterial and fungal communities was predicted using PICRUSt2 and FUNGuild tools, respectively (Toole et al., 2021; Zhang et al., 2022).

## 3 Results

### 3.1 Growth performance of *X. sorbifolium* at different densities and soil physicochemical properties

**Figure 1:**
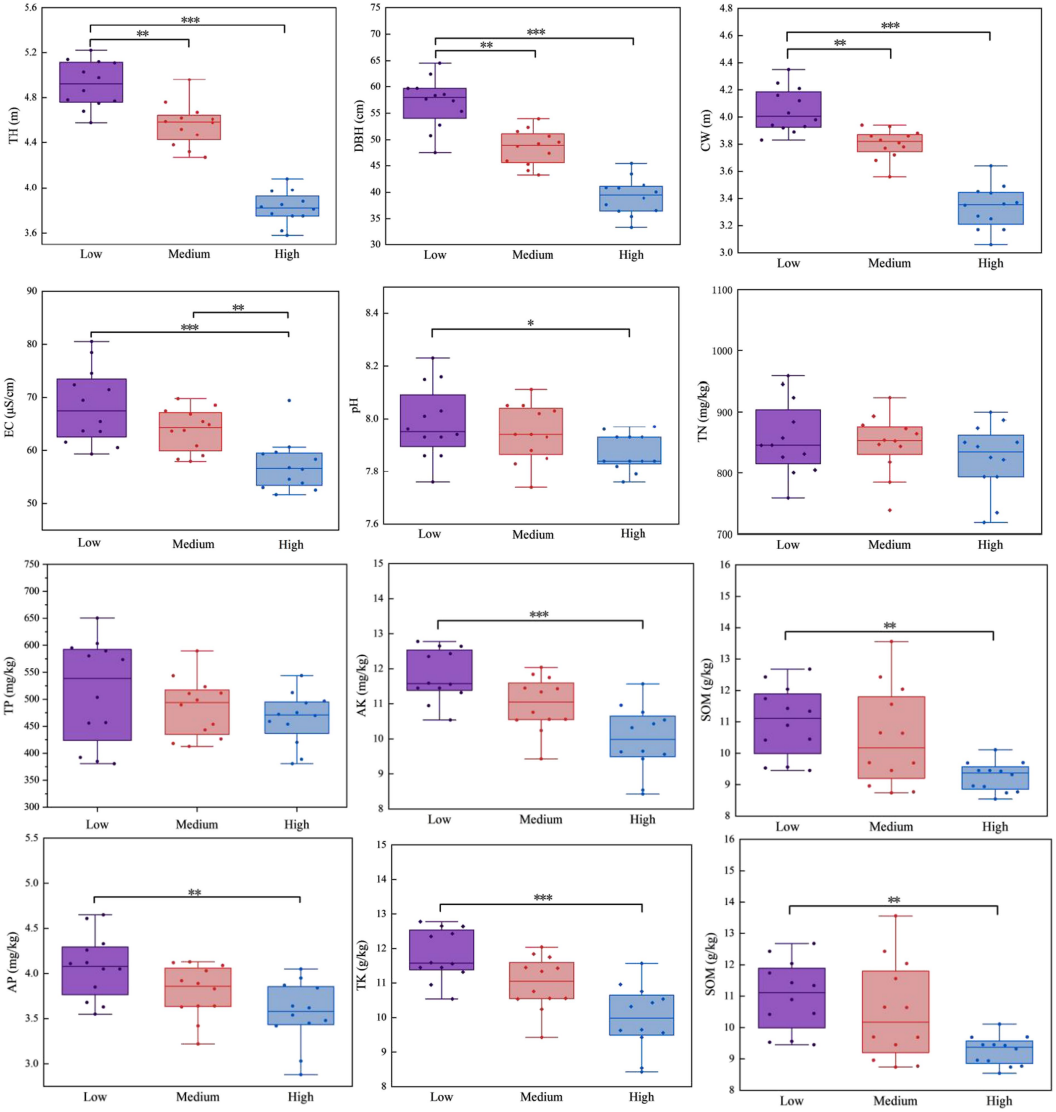
Growth indices and soil physicochemical properties of *X. sorbifolium* at different densities. Low, low density; Medium, medium density; High, high density. EC, electrical conductivity; TN, total nitrogen; TP, total phosphorus; TK, total potassium; AN, available nitrogen; AP, available phosphorus; AK, available potassium; SOM, soil organic matter; TH, tree height; DBH, diameter at breast height; CW, crown width. The symbols *, **, and *** indicate statistical significance levels at *P < 0.05, **P < 0.01, and ***P < 0.001, respectively.

Planting density had a significant effect on the growth of *X. sorbifolium* (Figure 1). With increasing planting density, TH, DBH, and CW all decreased significantly (*p* < 0.05). Two-way ANOVA (Table 1) showed that density had highly significant effects on TH, DBH, and CW (*p* < 0.001), while the provenance main effect and its interaction with density were not significant for TH and DBH (*p* > 0.05). However, a significant density × provenance interaction was observed for CW (*p* < 0.05). The TH of YC ranged from 3.81 to 4.96 m, and that of KL ranged from 3.84 to 4.87 m; the DBH of YC ranged from 38.03 to 57.18 cm, and that of KL ranged from 40.30 to 56.88 cm; the CW of YC ranged from 3.25 to 4.1 m, and that of KL ranged from 3.41 to 4.0 m. Under the same density, YC exhibited better growth performance than KL.

**Table 1:** Different provenances of *X. sorbifolium* in different densities of growth indicators and soil physicochemical properties.

Parameters	Low density		Medium density		High density		D	P	D × P
	YC	KL	YC	KL	YC	KL			
EC (μS/cm)	62.36 ± 2.06a	74.07 ± 4.01a	64.01 ± 3.98a	63.74 ± 3.06b	56.19 ± 2.20b	58.13 ± 5.62b	**	*	*
pH	8.03 ± 0.15a	7.94 ± 0.09a	7.89 ± 0.09a	8.00 ± 0.09ab	7.87 ± 0.06a	7.87 ± 0.07a	ns	ns	ns
TN (mg/kg)	870.49 ± 41.04a	842.79 ± 39.96a	861.12 ± 35.36a	833.70 ± 42.36a	835.53 ± 58.07a	812.87 ± 47.45a	ns	ns	ns
TP (mg/kg)	584.48 ± 35.55a	444.19 ± 43.63a	479.64 ± 34.38b	490.42 ± 62.98a	443.97 ± 43.54b	483.61 ± 44.60a	**	*	**
TK (mg/kg)	12.20 ± 0.49a	11.42 ± 0.60a	11.79 ± 0.76a	10.69 ± 0.73a	10.49 ± 0.74b	9.48 ± 0.76b	**	**	ns
AN (mg/kg)	47.40 ± 2.80a	43.31 ± 2.01a	48.43 ± 2.21a	41.35 ± 2.66ab	41.36 ± 2.00b	39.11 ± 3.14b	**	**	*
AP (mg/kg)	4.34 ± 0.23a	3.81 ± 0.21a	4.03 ± 0.09b	3.56 ± 0.19ab	3.75 ± 0.22b	3.38 ± 0.23b	**	**	ns
AK (mg/kg)	194.67 ± 21.42a	150.91 ± 17.39a	169.95 ± 19.99ab	155.82 ± 21.26a	148.47 ± 19.17b	168.48 ± 15.62a	**	ns	**
SOM (g/kg)	11.87 ± 0.60a	10.13 ± 0.68a	11.81 ± 1.02a	9.85 ± 0.38ab	9.29 ± 0.48b	9.21 ± 0.33b	**	**	*
TH (m)	4.96 ± 0.19a	4.87 ± 0.19a	4.59 ± 0.20b	4.54 ± 0.16b	3.81 ± 0.11c	3.84 ± 0.16c	***	ns	ns
DBH (cm)	57.18 ± 5.72a	56.88 ± 3.11a	49.11 ± 3.77b	47.83 ± 2.43b	38.03 ± 3.41c	40.30 ± 2.85c	***	ns	ns
CW (m)	4.10 ± 0.16a	4.00 ± 0.13a	3.77 ± 0.11b	3.83 ± 0.08a	3.25 ± 0.12c	3.41 ± 0.16b	***	ns	*

Values are presented as mean ± SD (*n* = 6). Different lowercase letters indicate significant differences among densities within each provenance (two-way ANOVA followed by Duncan’s multiple range test, *P* < 0.05). D, density effect; P, provenance effect; D × P: interaction effect. Significance levels: *p < 0.05, **P < 0.01, ***P < 0.001; ns, not significant.

Regarding soil physicochemical properties, the contents of EC, TK, AN, AP, AK, and SOM were significantly higher in low-density plantations than in high-density plantations (*p* < 0.05) (Figure 1). Two-way ANOVA (Table 1) revealed that density significantly affected EC, TP, TK, AN, AP, AK, and SOM (*p* < 0.01), but had no significant effect on pH or TN. Provenance significantly affected EC, TP, TK, AN, AP, and SOM (*p* < 0.05), and significant density × provenance interactions were detected for EC, TP, AN, AK, and SOM (*p* < 0.05). Between the two provenances, EC and pH were higher in KL soil than in YC soil, while the contents of TN, TP, TK, AN, AP, AK, and SOM were higher in YC soil than in KL soil.

In conclusion, low planting density resulted in better growth performance and higher soil nutrient levels in *X. sorbifolium*, and under the same density, the YC provenance outperformed the KL provenance.

### 3.2 Soil microbial community composition and diversity of *X. sorbifolium* at different densities

**Figure 2:**
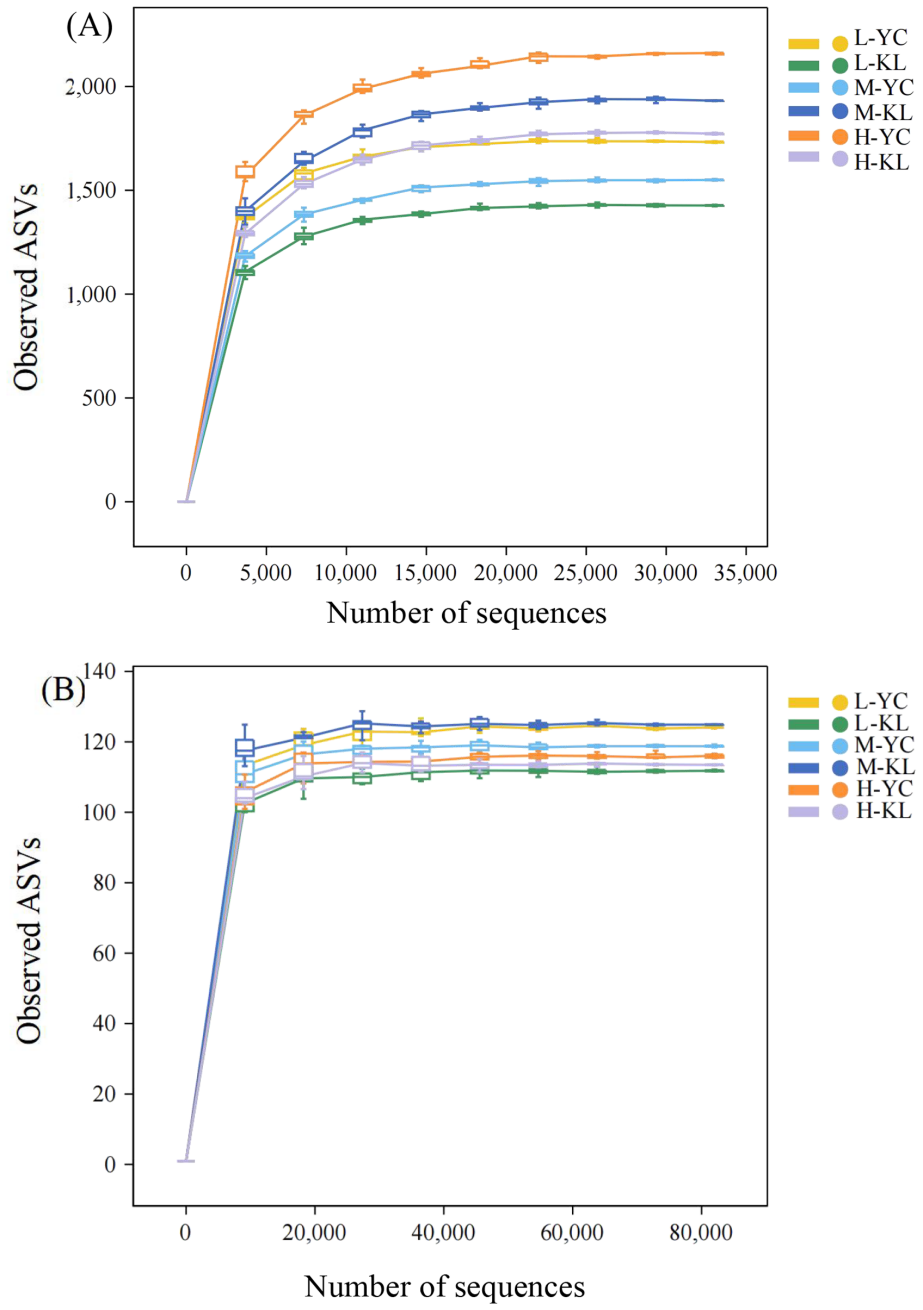
Rarefaction curves of soil bacterial **(A)** and fungal **(B)** communities under different planting densities.

A total of 1,243,583 high-quality bacterial sequences (average 34,544 sequences per sample) and 2,436,844 high-quality fungal sequences (average 67,690 sequences per sample) were obtained from 36 soil samples using the Illumina MiSeq platform. As shown in Figure 2, the rarefaction curves for all bacterial and fungal samples reached plateau phases, indicating that the sequencing depth was sufficient and the sampling of bacterial and fungal community diversity was effective for subsequent analyses. To ensure the validity and accuracy of the analytical results, all samples were rarefied based on the minimum sequence number prior to data analysis. After rarefaction, 34,523 bacterial sequences and 42,098 fungal sequences per sample were retained for subsequent analyses.

**Figure 3:**
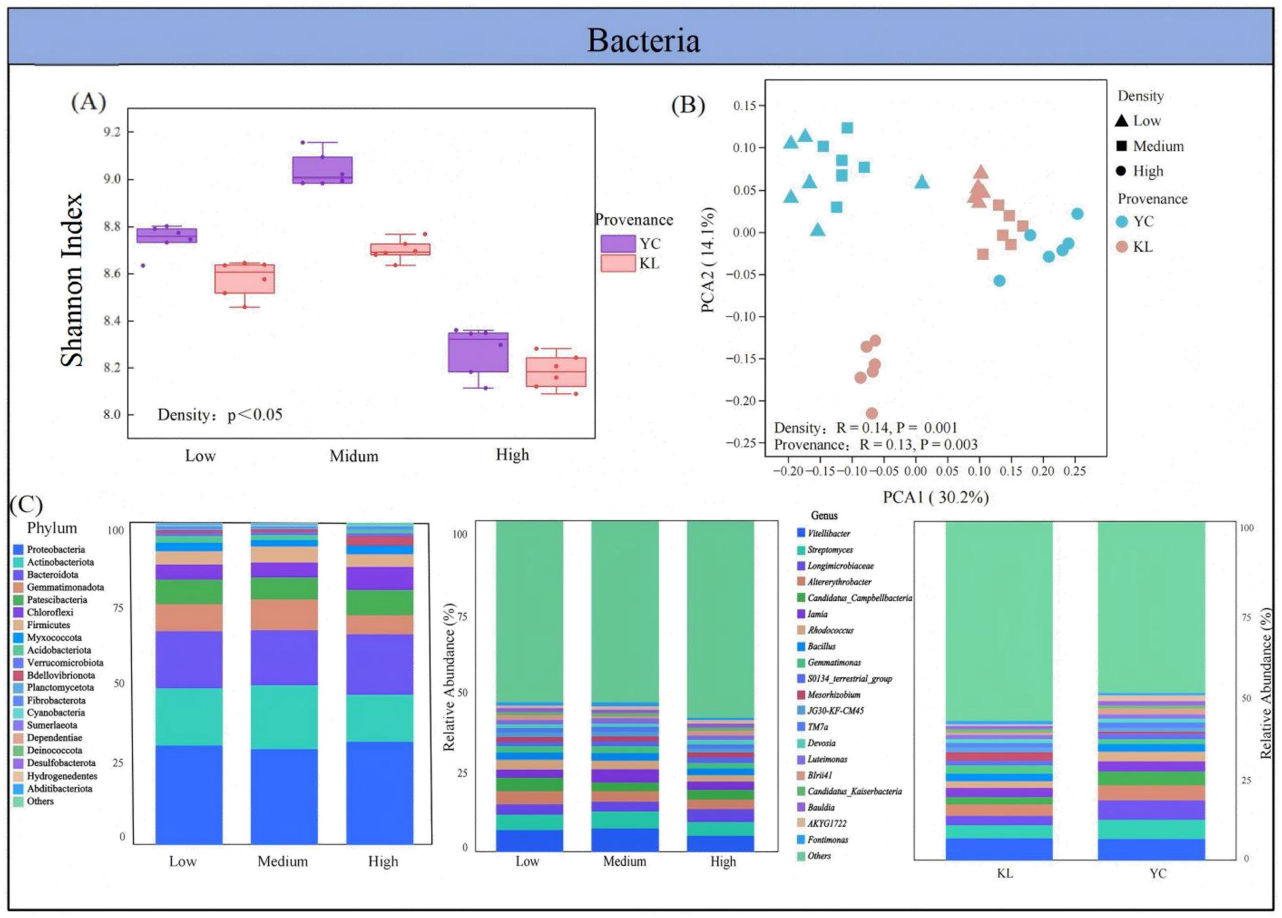
Composition and diversity of soil bacterial communities: **(A)** Shannon diversity index; **(B)** principal coordinate analysis (PCoA) based on Bray-Curtis distance; **(C)** stacked percentage abundance chart of dominant bacterial phyla and genera.

**Figure 4:**
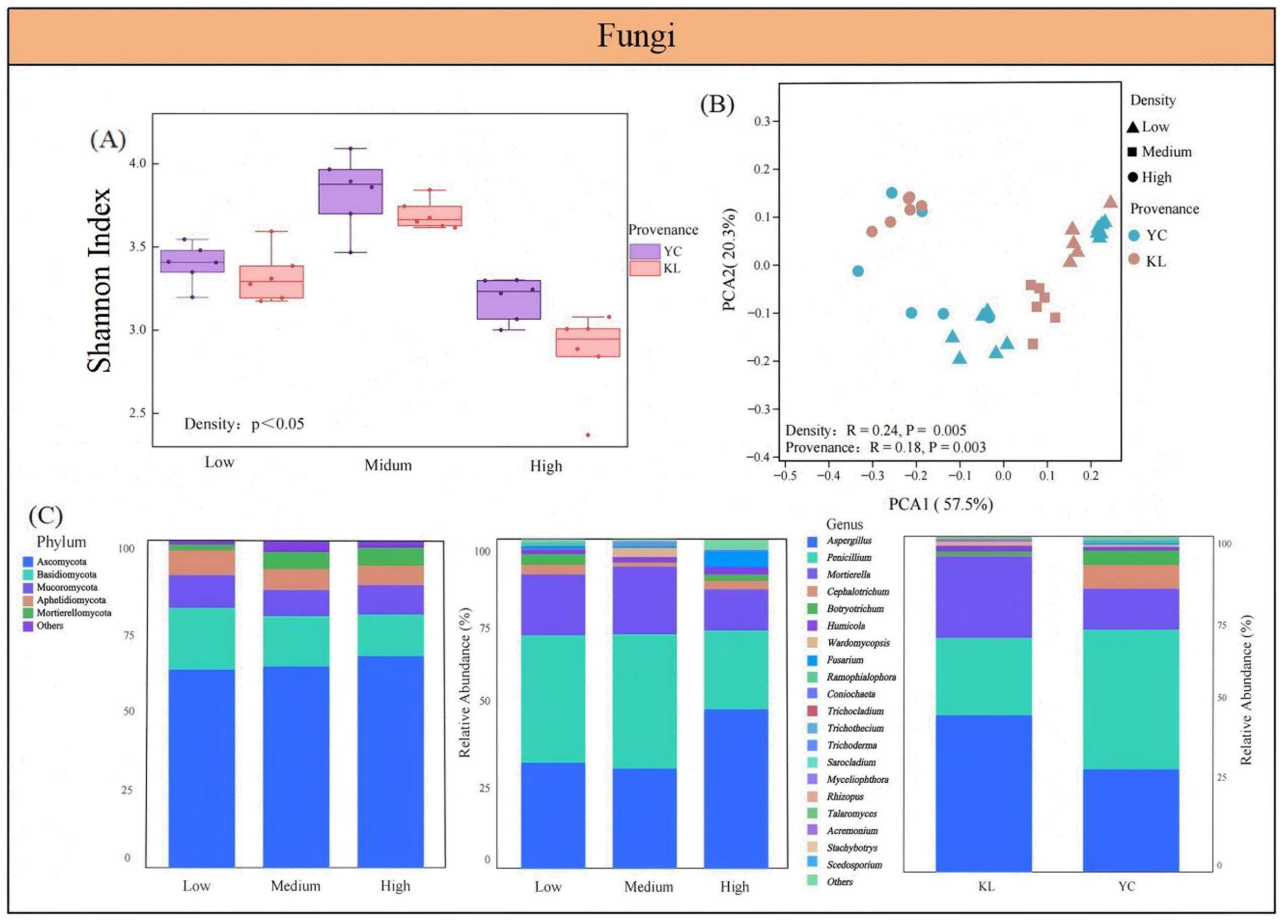
Composition and diversity of soil fungal communities: **(A)** Shannon diversity index; **(B)** principal coordinate analysis (PCoA) based on Bray-Curtis distance; **(C)** stacked percentage abundance chart of dominant fungal phyla and genera.

The Shannon indices for soil bacteria and fungi from both provenances of *X. sorbifolium* were highest at medium density, followed by low and high density (Figures 3A, 4A). The Shannon index of bacterial communities (8.18–9.17) was higher than that of fungal communities (2.68–4.31). In different densities, the Shannon indices of soil bacteria and fungi in YC were higher than those in KL. PCoA results indicated that the composition of soil microbial communities of *X. sorbifolium* from different densities and provenances had significant differences (*p* < 0.05) (Figures 3B, 4B). PERMANOVA revealed that density had a greater impact on bacterial communities (*R*^2^ = 0.14) and fungal communities (*R*^2^ = 0.24) than provenance (*R*^2^ = 0.13, *R*^2^ = 0.18).

In three soil densities, the main bacterial phyla were Proteobacteria, Actinobacteriota, Bacteroidota, Gemmatimonadota, Patescibacteria, and Chloroflexi, among others (Figure 3C). The dominant bacterial genera were *Vitellibacter*, *Streptomyces*, *Longimicrobiaceae*, *Altererythrobacter*, and *Candidatus-Campbellbacteria*. Potentially beneficial bacteria included *Streptomyces*, *Altererythrobacter*, *Rhodococcus*, *Bacillus*, and *Luteimonas*. Additionally, beneficial fungi were significantly more abundant in low-density and medium-density soils compared to high-density soils. In the two provenances: *Streptomyces*, *Altererythrobacter*, and *Rhodococcus* were higher in YC soil than in KL.

In three soil densities, the main fungal phyla were Ascomycota, Basidiomycota, Mucoromycota, Aphelidiomycota, and Mortierellomycota (Figure 4C). The main fungal genera were *Aspergillus*, *Penicillium*, *Mortierella*, *Cephalotrichum*, etc. Potentially beneficial fungi included *Penicillium*, *Humicola*, *Ramophialophora*, and *Trichoderm*a. Potentially pathogenic fungi included *Aspergillus*, *Mortierella*, *Botryotichum*, *Fusarium*, *Rhizopus*, and *Stachybotrys*. The combined relative abundance of the *Aspergillus* and *Penicillium* genera exceeds 50% in all three soil densities, making them dominant genera. In low-density and medium-density soils, *Penicillium* was significantly higher than in high-density soils, while in high-density soils, *Aspergillus* is higher than in low-density and medium-density soils. Regarding provenance differences, YC soils exhibited a higher relative abundance of *Penicillium*, whereas KL soils had higher abundances of *Aspergillus* and *Mortierella*.

### 3.3 Assembly process of soil microorganisms in *X. sorbifolium* soils of different densities

**Figure 5:**
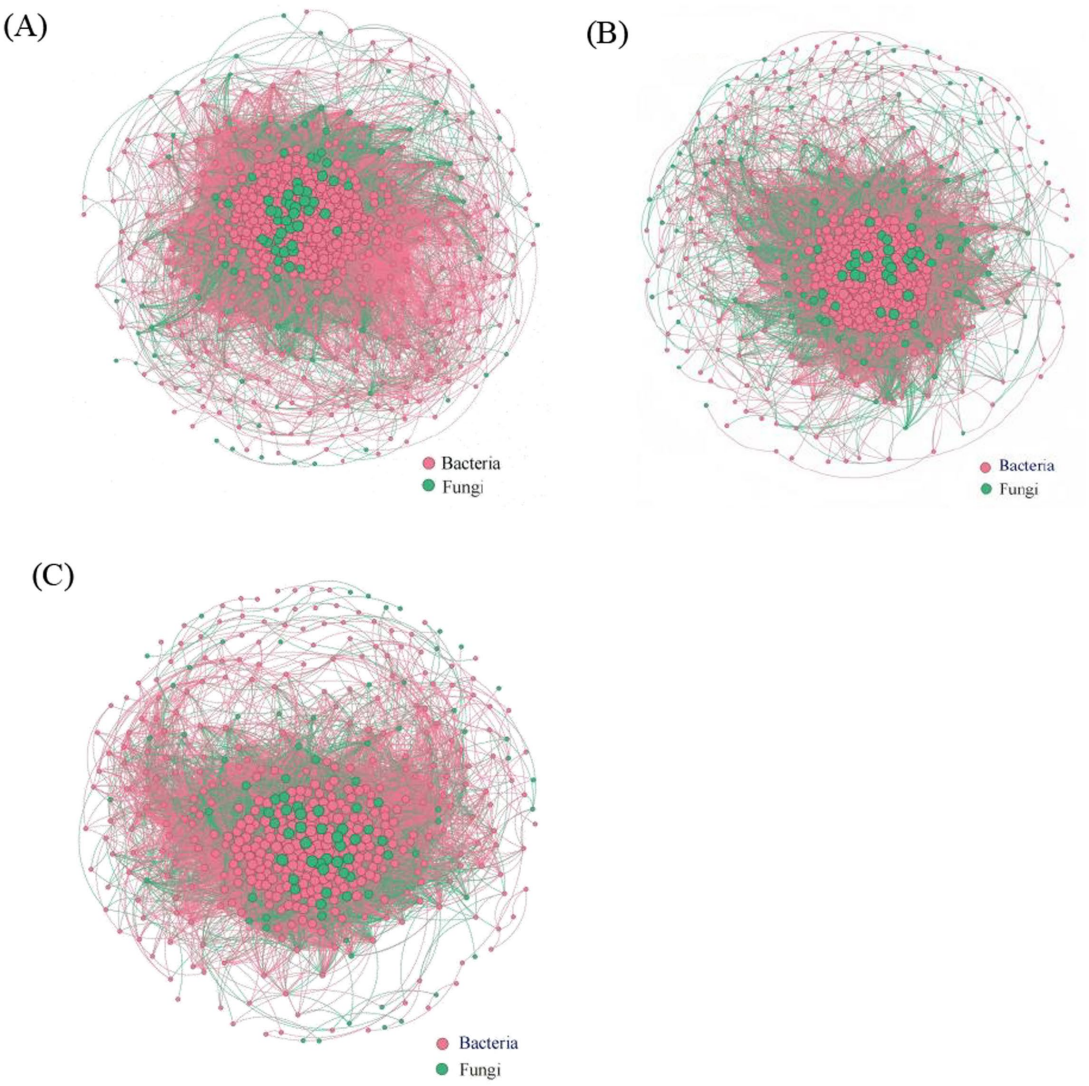
Co-occurrence networks of soil microbial communities under different planting densities. **(A)** Low density; **(B)** Medium density; **(C)** High density.

We constructed microbial co-occurrence networks under different forest stand densities (Figure 5) and analyzed their topological properties (Table 2). The results showed that the microbial network in low-density soil was the most complex, with the highest number of nodes (625) and edges (14,500), as well as the highest average degree (46.4) and network density (0.074). The network in medium-density soil exhibited the highest clustering coefficient (0.63) and the shortest average path length (3.84). In contrast, the network in high-density soil had the largest network diameter (12) and the highest proportion of negative correlations.

**Table 2:** Topological parameters of soil microbial co-occurrence networks in *X. sorbifolium* soils of different densities.

Microorganisms across different density	Nodes_number	Edges_number	Average_degree	Average_path_length	Clustering_coefficient	Network_diameter	Positive.cor_num	Negative.cor_num	Network_density
Low	625	14,500	46.4	3.52	0.67	9	9,500	5,000	0.074
Medium	572	11,800	41.3	3.84	0.63	10	7,500	4,300	0.072
High	503	7,800	31.0	4.35	0.56	12	4,800	3,000	0.062

Low, low density; Medium, medium density; High, high density.

**Figure 6:**
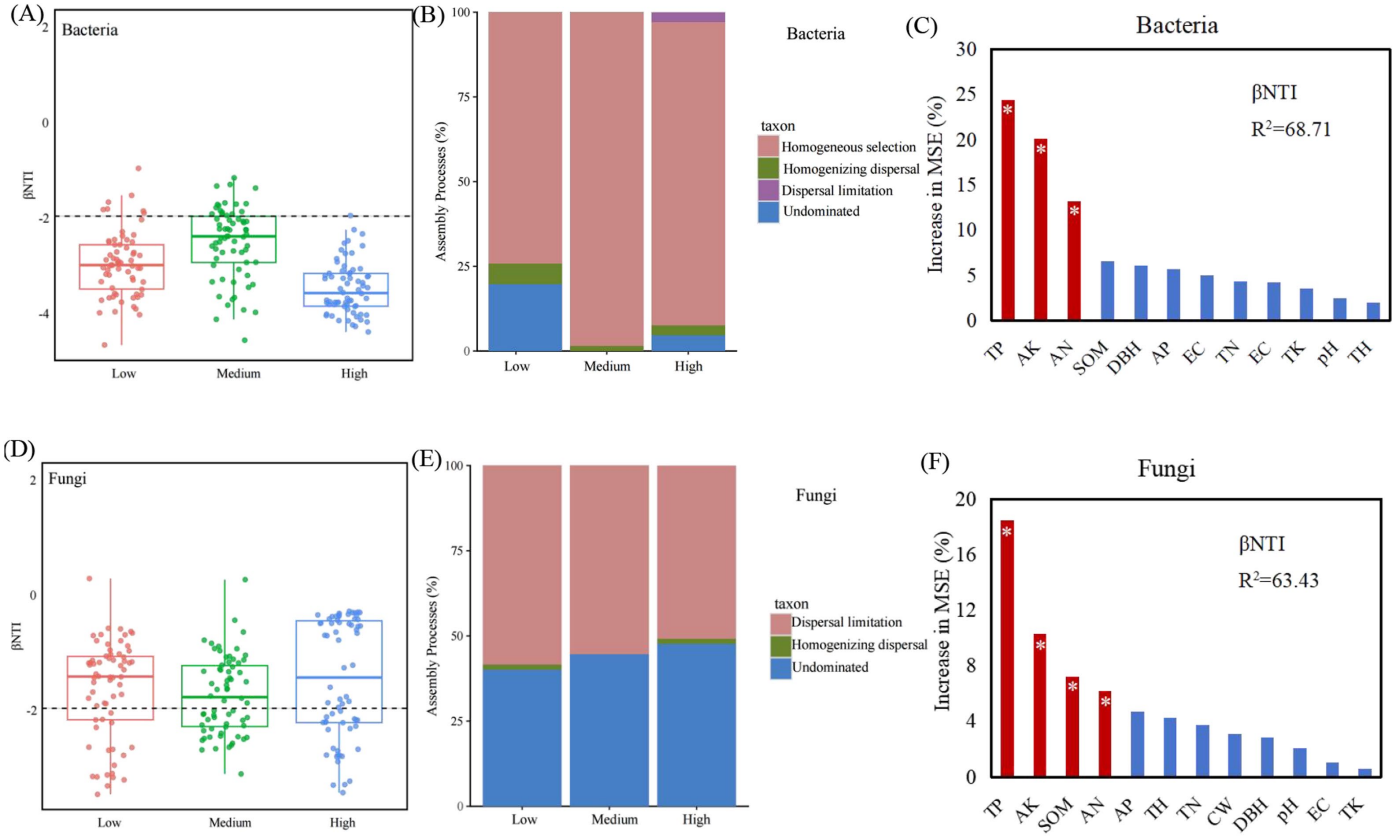
Analysis of soil microbial community assembly processes and key driving factors under different planting densities. **(A,B)** Assembly mechanisms **(A)** and key environmental drivers **(B)** of bacterial communities. Values of |βNTI| > 2 indicate deterministic assembly, while |βNTI| < 2 indicate stochastic assembly. Homogeneous selection: deterministic process; Dispersal limitation, Homogenizing dispersal, and undominated: stochastic processes. **(C,D)** Assembly mechanisms **(C)** and key environmental drivers **(D)** of fungal communities. Low: low density, medium: medium density, high: high density.

The results showed that the dominant mechanisms governing the assembly processes of fungal and bacterial communities at different densities differed (Figure 6). Bacterial community assembly was primarily determined by deterministic processes (βNTI < −2), while fungal community assembly was largely the result of a random process (2 > βNTI > − 2). Among these, the primary process governing bacterial community assembly was Homogeneous selection (87.37%), followed by Undominated (8.07%), Homogenising dispersal (3.53%), and Dispersal limitation (3.00%) (Figure 6A). Fungal communities are influenced by Undominated (61.1%), Homogeneous selection (37.9%), and Homogenising dispersal (1.00%) (Figure 6B).

Environmental factors have different effects on microbial community assembly. Among them, TP, AK, and AN play a key role in bacterial communities (Figure 6C). TP, AK, SOM, and AN are crucial for fungal communities (Figure 6D).

### 3.4 Effect of environmental factors on microbial communities

**Figure 7:**
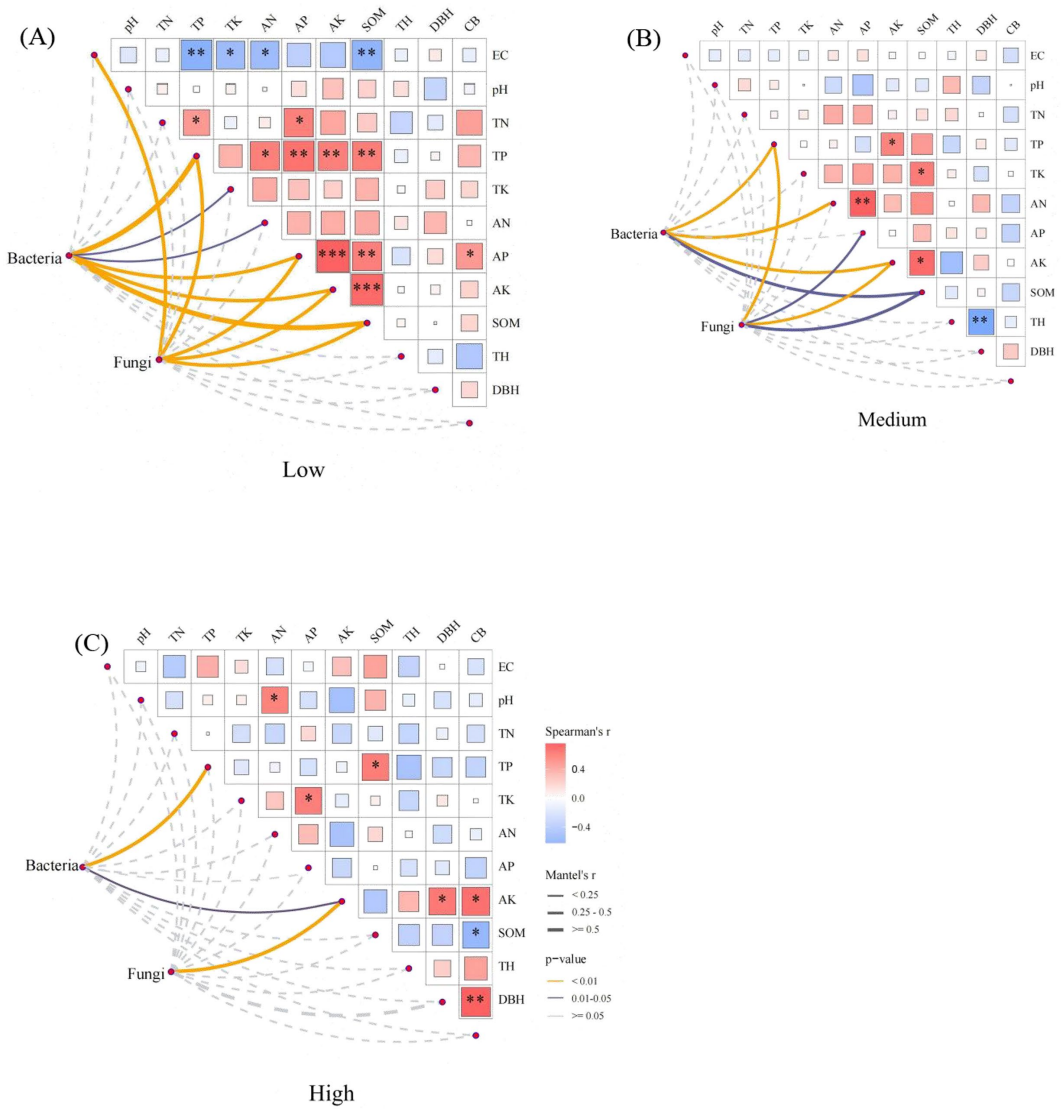
Mantel test analysis of correlations between microbial communities and soil environmental factors under different densities **(A–C)**. TP, total phosphorus; AK, available potassium; SOM, soil organic matter; TN, total nitrogen; AN, available nitrogen; AP, available phosphorus; EC, electrical conductivity.

**Figure 8:**
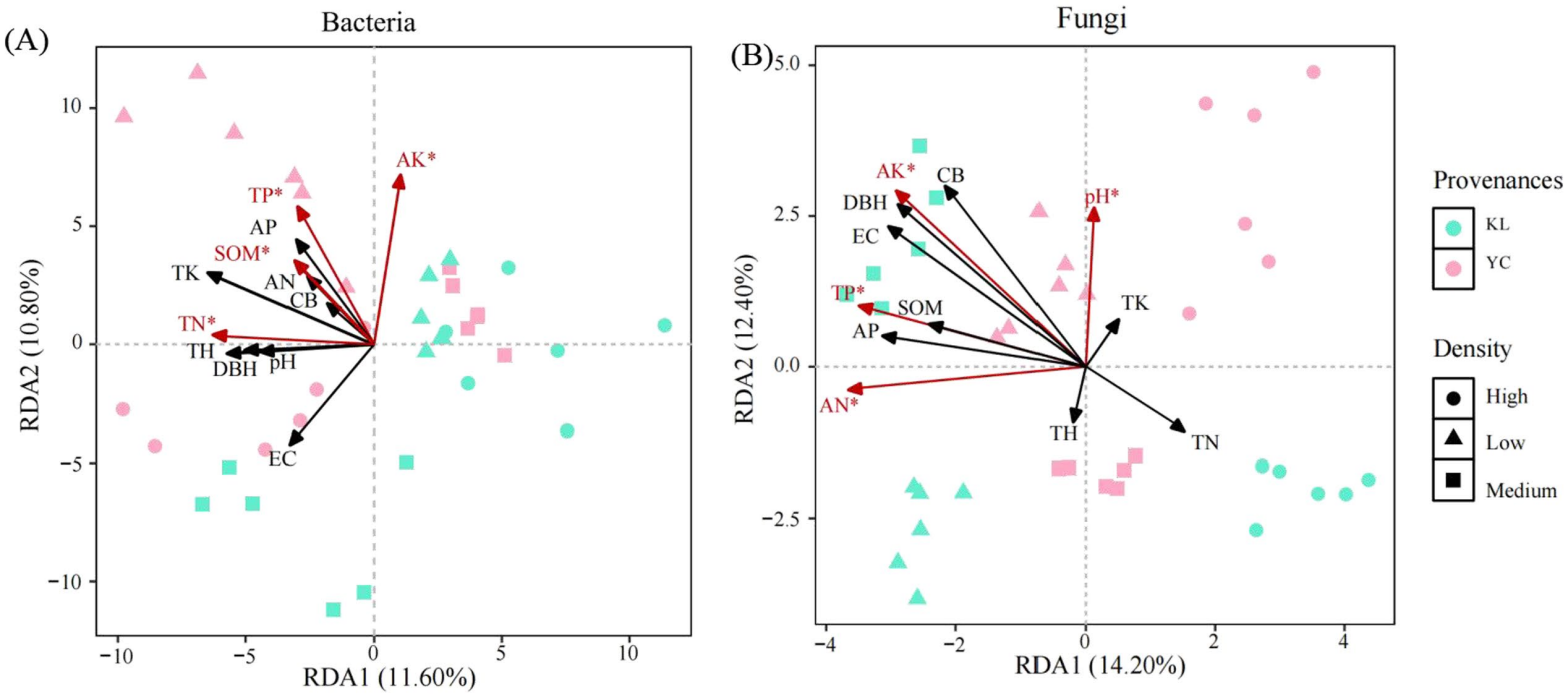
Redundancy analysis (RDA) of soil microbial communities and environmental factors: **(A)** bacterial communities; **(B)** fungal communities. EC, electrical conductivity; TN, total nitrogen; TP, total phosphorus; TK, total potassium; AN, available nitrogen; AP, available phosphorus; AK, available potassium; SOM, soil organic matter; TH, tree height; DBH, diameter at breast height; CW, crown width.

In low density, bacterial community showed highly significant correlation with soil TP, AP, AK and SOM (*p* < 0.01), and significant correlation with TK and AK (*p* < 0.05), and fungal community showed highly significant correlation with soil EC, TP, AP, AK and SOM (*p* < 0.01) (Figure 7A). In medium-density soil, bacterial communities showed extremely significant correlations with soil AN, TP, and AK (*p* < 0.01) and significant correlations with SOM (*p* < 0.05); fungal communities showed extremely significant correlations with soil TP and AK (*p* < 0.01) and significant positive correlations with AP and SOM (*p* < 0.05) (Figure 7B). In high-density soils, bacterial communities showed extremely significant correlations with soil TP (*p* < 0.01) and significant correlations with soil AK (*p* < 0.05), while fungal communities showed extremely significant correlations with soil AK (*p* < 0.01) (Figure 7C). RDA results showed that the first axis of bacterial communities explained 11.60% of the variance, and the second axis explained 10.80%, with both axes together explaining 22.40% of the total variance. The first axis of the fungal community explained 14.20% of the variance, and the second axis explained 12.40%, with both axes together explaining 26.60% of the total variance (Figure 8). Among these, TP, AK, SOM, and TN were the primary factors influencing the bacterial community (Figure 8A), while AK, TP, AN, and pH were the primary factors driving the fungal community (Figure 8B).

### 3.5 Prediction and analysis of soil microbial community function

**Figure 9:**
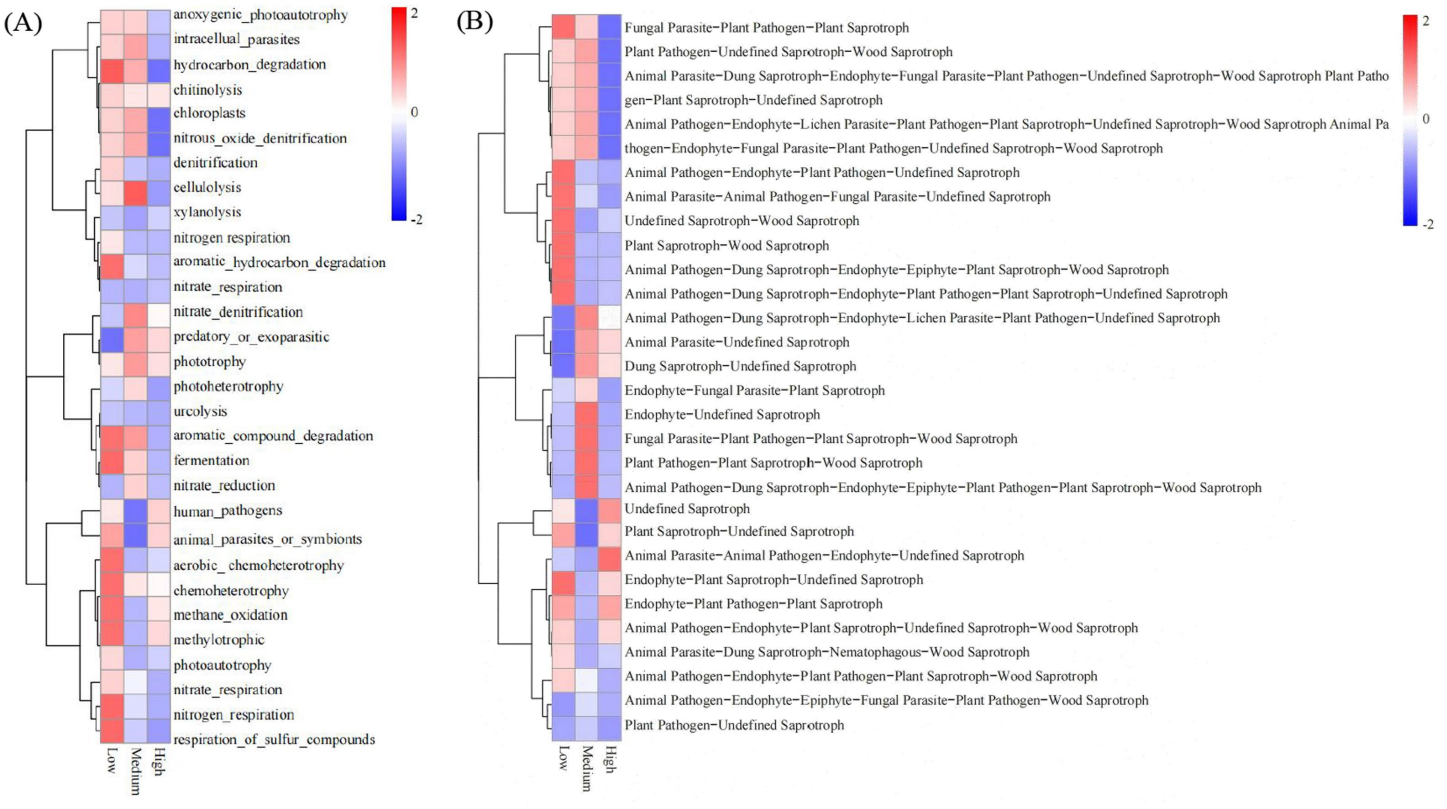
Functional predictions of bacterial **(A)** and fungal **(B)** communities under different planting densities.

As shown in Figure 9A, hydrocarbon degradation, aromatic hydrocarbon degradation, aromatic compound degradation, fermentation, aerobic chemoheterotrophy, chemoheterotrophy, methylotrophic, nitrogen respiration, and respiration of sulfur compounds were significantly enriched in low-density soils. Cellulolysis was significantly enriched in medium-density soil. As shown in Figure 9B, low-density and high-density soils host a wide range of functional types, from fungal parasites, endophytic fungi, animal pathogens, and plant pathogens to plant saprophytes, wood saprotrophs, and undefined saprophytes. This indicates that low-density and high-density soil fungal communities exhibit high diversity and complexity.

## 4 Discussion

### 4.1 Effects of stand density on the growth of *X. sorbifolium* and soil physicochemical properties

Stand density can affect the level of nutrient acquisition by individual trees and indirectly influence soil physical and chemical properties by regulating canopy cover, tree growth space, and the microenvironment within the forest (Zhang J. et al., 2021; Zhang Y. X. et al., 2021). In this study, stand density had a significant effect on the growth and physical and chemical properties of *X. sorbifolium*. As stand density increased, the tree height, breast height diameter, and crown spread of *X. sorbifolium* all showed a decreasing trend. At higher stand densities, intensified competition for soil nutrients, water, and light leads to reduced growing space. Consequently, individual *X. sorbifolium* trees exhibit weaker growth, manifested as slower height and diameter growth and a smaller crown spread. This conclusion is similar to the findings in poplar, indicating that an appropriate reduction of the stand density is beneficial for the growth of *X. sorbifolium* and the cultivation of large-diameter timber (Qiao et al., 2024). Compared to high-density soils, the EC, pH, AK, AN, AP, TK, and SOM content in low-density soils were significantly higher. In low-density artificial forests, larger crown gaps can enhance light penetration, enzyme activity, and forest transpiration, thereby promoting litter decomposition and nutrient cycling. Additionally, reduced competition between trees allows nutrients to be distributed more evenly in the soil, thus improving soil fertility (Wang et al., 2021).

### 4.2 The effect of stand density on the composition and diversity of soil microbial communities in *X. sorbifolium*

Soil nutrient levels are an important factor influencing microbial communities. Soils with high nutrient levels typically provide abundant resources, promoting microbial growth and reproduction, thereby enhancing the stability of microbial communities (Wang et al., 2024). In this experiment, low-density and medium-density soils with high nutrient levels provided an optimal environment for microbial survival, with Shannon indices for low-density and medium-density soils exceeding those of high-density soils. In high-density soils, available nutrients for microorganisms were limited, and soil pH decreased, with soil acidification becoming increasingly unfavorable for the growth of most microorganisms (Xie et al., 2023). These factors led to changes in microbial community diversity and composition. In this study, the potential beneficial bacteria *Streptomyces*, Altererythrobacter, Rhodococcus, and Bacillus were significantly higher in low-density and medium-density soils than in high-density soils. *Streptomyces* can produce plant growth hormones, which promote root development, increase plant biomass, and enhance fruit yield. Additionally, they possess nitrogen-fixing capabilities, converting atmospheric nitrogen into plant-available nitrogen, thereby enhancing soil fertility (Newitt et al., 2019). The increase in Altererythrobacter abundance helps maintain the balance of the soil microbial community, promotes the proliferation of other beneficial bacteria, and thereby enhances the overall functionality of the soil (Zhang et al., 2019). Beneficial bacteria play a crucial role in soil ecosystems, as they can promote plant growth, improve soil structure, and enhance soil fertility. Their presence highlights the positive impact of low-density and medium-density planting on soil microbial communities. Rhodococcus excels in degrading toxic pesticides and other pollutants in soil, with most strains exhibiting significant growth-promoting and disease-resistant properties (Kim et al., 2018). Bacillus is one of the most common beneficial bacteria in agriculture, capable of improving soil fertility through processes such as phosphorus solubilization, potassium mobilization, and nitrogen fixation, and can produce various antimicrobial substances to inhibit the growth of plant pathogens (Zheng et al., 2021). Compared to low-density and medium-density soils, high-density soils have a higher relative abundance of potential pathogens such as *Aspergillus* and *Fusarium*. *Aspergillus* can produce toxins in soil, contaminating crops, and is the primary pathogen responsible for aflatoxin contamination in crops such as peanuts and corn (Lang, 2023). *Fusarium* can enter plants through root tips, lateral roots, or root wounds, and once it enters the xylem, it blocks the vessels, causing plant wilting. Diseases caused by *Fusarium* include wheat fusarium head blight and corn wilt disease (Wu et al., 2023). These results are similar to those showing that high-density planting in.

*Cunninghamia lanceolata* plantations leads to soil microecological imbalance, characterized by an increase in pathogenic bacteria and a decrease in beneficial bacteria (Zhu et al., 2021). This suggests that high-density planting may create opportunities for the spread and reproduction of pathogenic bacteria, which has adverse effects on soil health and plant growth (Cui et al., 2021).

### 4.3 Effects of different densities on soil microorganisms: interactions and key drivers

Differences in the density of artificial forests can alter the survival strategies and functional expression of soil microorganisms. These changes can promote positive interactions within microbial communities, thereby enhancing community stability. Meanwhile, they may trigger negative interactions between microbial populations as well, leading to community instability (Iddrisu et al., 2024). Microbial network analysis is widely applied in elucidating the mechanisms underlying microbial interactions in response to environmental disturbances (Xue et al., 2018). It has been found that compared to simple networks, complex networks with higher average clustering coefficients and average degrees, as well as shorter average path lengths are of greater stability and ecological resilience (Gao et al., 2022). This suggests that low- and medium-density stands are more conducive to maintaining the health and ecological stability of soil microbial communities. In addition, we found abundant mycorrhizal fungi and beneficial bacteria in low- and medium-density soils. Mycorrhizal fungi can form mycorrhizae with plant roots, promoting the growth and stress resistance of the plant while exerting a positive influence on other soil microorganisms (Fei et al., 2022). This positive interaction is of great significance for maintaining the stability of microbial communities, and the results of the microbial symbiotic network once again confirm this conclusion. Community assembly is jointly regulated by deterministic and stochastic processes. In the present study, soil bacterial communities at different densities were primarily dominated by deterministic homogeneous selection, while fungal communities were predominantly governed by stochastic processes. The assembly mechanisms of fungi and bacteria differ at both large and small scales. At a large scale, fungal community assembly is dominated by random homogeneous dispersal, while bacterial community assembly is governed by deterministic homogeneous selection (Wang et al., 2020). At a small scale, bacterial community assembly is dominated by random ecological drift, while fungal community assembly by random dispersal limitation (Cao et al., 2020). These differences are primarily due to the different life history strategies and niche widths of bacterial and fungal communities (Wu et al., 2018; Sheng et al., 2024). Mantel test and RDA results indicated that soil nutrients were closely related to microbial communities, with TP and AK being the primary drivers influencing microbial communities. Phosphorus and potassium contents in soil directly affect microbial metabolic activity and growth rates; adequate phosphorus and potassium levels can enhance microbial activity and promote microbial diversity (Li et al., 2024). The availability of TP and AK can influence microbial metabolic pathways, such as energy production, material cycling, and biosynthesis (Zhang et al., 2024). Random forest analysis results also showed that TP, AK, and AN played a key role in bacterial communities, while TP, AK, SOM, and AN were crucial for fungal communities. We therefore conclude that TP and AK are the overarching factors governing both microbial community structure and assembly.

### 4.4 Prediction and analysis of microbial functions in soils of different densities

Bacterial functional groups in soil play a crucial role in biogeochemical cycles, and there are differences in the relative abundance of bacterial functional groups in soils of varying densities. In this study, low-density soils were significantly enriched by aromatic compound degradation, aromatic hydrocarbon degradation, and hydrocarbon degradation, which are closely associated with the decomposition and metabolism of organic matter. Studies indicate that soils rich in aromatic compounds, hydrocarbons, and other organic substances provide abundant carbon sources and energy for specific degradation bacteria, thereby facilitating their significant enrichment (Georgiou et al., 2024). Compared to medium- and high-density soils, the chemoheterotrophic and aerobic chemoheterotrophic functional groups of low-density soils are higher in relative abundance, as these bacteria rely on the oxidation of organic matter to obtain carbon sources and nutrients (Xiao et al., 2018). Therefore, the growth and metabolic activities of them are relatively better maintained in nutrient-rich low-density soils. Medium-density soils exhibit significant enrichment in cellulolysis functions, possibly due to the abundance of plant residues and cellulose materials in the soil, which provide an optimal growth environment for cellulose-degrading bacteria. These bacteria contribute to the efficient degradation of cellulose, which is crucial for carbon cycling and soil fertility (Datta, 2024). Compared to low- and medium-density soils, methane oxidation and methylotrophic functions are weaker in high-density soils. This may be due to reduced nutrient levels and low organic carbon content in high-density soils, making it difficult for carbon-utilizing bacteria to obtain sufficient carbon sources such as methane and methanol for carbon cycling, thus limiting their functional activity and hindering their growth and reproduction (Yan et al., 2025; Yang et al., 2023).

The functional prediction results of soil fungal communities revealed that low- and medium-density soil fungal communities exhibited extensive functional diversity, encompassing a wide range of functional types, including fungal parasites, endophytic fungi, animal pathogens, plant pathogens, plant saprophytes, and undefined saprophytes, with various ecological roles. The broad distribution of ecological roles highlights the uniqueness and richness of low-density and medium-density soil environments (Lu et al., 2024; Cao, 2025). High functional diversity typically indicates a more stable and resilient ecosystem with better adaptability to environmental changes and disturbances (Husson, 2013), which suggests that low- and medium-density soils may possess more robust fungal stability. In high-density soils, the abundance of saprophytic nutrient-type fungi and certain functional groups (undefined saprophytes, plant pathogens, parasitic fungi-undefined saprophytes, etc.) were relatively low. Saprophytic fungi primarily obtain their nutrients from SOM, which decomposes organic matter such as plant residues and animal manure into mineral nutrients that plants can absorb and utilize (Mattila, 2024; Peng et al., 2022). In this study, as the planting density of *X. sorbifolium* increased, the soil SOM content gradually decreased, which may be the reason for the lower abundance of saprophytic nutrient-type fungi and other relevant groups in high-density soils. This study employed high-throughput sequencing to predict the functions of bacteria and fungi. However, the limited depth of information provided by this technique leads to certain inherent limitations in the prediction results. Future research will integrate transcriptomic and proteomic analyses to enable a more in-depth validation and exploration of microbial functions.

## 5 Conclusion

Our findings indicate that lower planting density treatments of *X. sorbifolium* promote plant growth and soil nutrient regulation, while also increasing soil microbial community diversity. Moreover, an increased abundance of potentially beneficial bacteria plays a crucial role in maintaining a more stable and complex soil ecosystem structure. Soil TP and AK were identified as key factors influencing the microbial communities, primarily by driving the assembly processes of both bacterial and fungal communities. Furthermore, we employed PICRUSt2 and FUNGuild to predict the potential functions of the bacterial and fungal communities under different density conditions. These results provide a theoretical basis for a deeper understanding of the composition and distribution of soil microbial communities and their driving mechanisms across varying forest stand densities.
